# Phospholipase D1 Mediates TNFα-Induced Inflammation in a Murine Model of TNFα-Induced Peritonitis

**DOI:** 10.1371/journal.pone.0010506

**Published:** 2010-05-05

**Authors:** Swaminathan Sethu, Peter N. Pushparaj, Alirio J. Melendez

**Affiliations:** 1 Department of Physiology, Yong Loo Lin School of Medicine, National University of Singapore, Singapore, Singapore; 2 Division of Immunology, Infection and Inflammation, Glasgow Biomedical Research Centre, University of Glasgow, Glasgow, Scotland, United Kingdom; University of Georgia, United States of America

## Abstract

**Background:**

Tumor Necrosis Factor alpha (TNFα) is a pleiotropic cytokine extensively studied for its role in the pathogenesis of a variety of disease conditions, including in inflammatory diseases. We have recently shown that, *in vitro*, that TNFα utilizes PLD1 to mediate the activation of NFκB and ERK1/2 in human monocytes. The aim of this study was to investigate the role(s) played by phospholipase D1 (PLD1) in TNFα-mediated inflammatory responses *in vivo*.

**Methodology/Findings:**

Studies were performed *in vivo* using a mouse model of TNFα-induced peritonitis. The role of PLD1 was investigated by functional genomics, utilizing a specific siRNA to silence the expression of *PLD1*. Administration of the siRNA against PLD1 significantly reduced PLD1 levels *in vivo*. TNFα triggers a rapid pyrogenic response, but the *in vivo* silencing of PLD1 protects mice from the TNFα-induced rise in temperature. Similarly TNFα caused an increase in the serum levels of IL-6, MIP-1α and MIP-1β: this increase in cytokine/chemokine levels was inhibited in mice where PLD1 had been silenced. We then induced acute peritonitis with TNFα. Intraperitoneal injection of TNFα triggered a rapid increase in vascular permeability, and the influx of neutrophils and monocytes into the peritoneal cavity. By contrast, in mice where PLD1 had been silenced, the TNFα-triggered increase in vascular permeability and phagocyte influx was substantially reduced. Furthermore, we also show that the TNFα-mediated upregulation of the cell adhesion molecules VCAM and ICAM1, in the vascular endothelium, were dependent on PLD1.

**Conclusions:**

These novel data demonstrate a critical role for PLD1 in TNFα-induced inflammation *in vivo* and warrant further investigation. Indeed, our results suggest PLD1 as a novel target for treating inflammatory diseases, where TNFα play key roles: these include diseases ranging from sepsis to respiratory and autoimmune diseases; all diseases with considerable unmet medical need.

## Introduction

Tumor Necrosis Factor alpha (TNFα) is a pleiotropic cytokine extensively studied for its role in the pathogenesis of a variety of disease conditions, which is known to have a wide range of beneficial and deleterious effects in humans [1], [2]. TNFα is produced by a variety of cells which include: macrophages, monocytes, lymphocytes, NK cells, eosinophils, keratinocytes, langerhan cells, kupffer cells, glial cells, adipocytes and fibroblasts [1]–[3]. This cytokine is known to be produced in response to a wide range of stimuli such as, bacterial toxins (e.g. LPS); infections (bacterial, viral, fungal, mycobacterial and parasitic); antigen-antibody complexes; injury; host inflammatory agents (products of the complement activation, auto-antibodies and cytokines); as well as toxic and non-toxic environmental challenges [1], [3]. TNFα elicits a wide spectrum of cellular responses which mediates inflammation, regulates immune response and also induces apoptosis in certain types of cancer cells [4], [5]. Appropriate levels of TNFα are necessary for homeostatic functions like protection from infection, haematopoiesis, immune response regulation, cellular growth in wound healing, tumor regression and immune surveillance [6]. In contrast, dysregulation in TNFα production or signaling has been associated with a wide range of inflammatory disorders, ranging from sepsis to anaphylaxis to autoimmune diseases [1], [2], [4], [6]–[8]. TNFα mediates its inflammatory functions by inducing the production of various proinflammatory cytokines and chemokines, activation of leukocytes and lymphocytes, inducing vascular permeability, enhancing the expression of adhesion molecules in immune cells as well as in the vascular endothelium, and promoting inflammatory cell migration, proliferation and differentiation [1]–[3], [5], [9]. Therefore, it is not surprising that much effort has been directed at blocking TNFα in human diseases; however, with mixed success [8], [9]. Incidentally, in spite of a great body of literature on the inflammatory pathways triggered by TNFα in various cell types, no significant validation of potential signaling targets has been documented.

We recently reported that in human monocytes, TNFα activates the Phosphatidylcholine-specific Phospholipase D1 (PLD1), and showed that inhibition of PLD-generated active products, or genetic-silencing of PLD1, largely inhibits TNFα-triggered key intracellular signaling pathways pivotal in the TNFα-mediated proinflammatory responses, suggesting a potential role for PLD1 in TNFα-mediated inflammation [10].

Phosphatidylcholine (PC), in addition to being a structural constituent of cell membranes, is a source of important signaling molecules. In particular, PC-derived phosphatidic acid (PA) and diacylglycerol (DAG) have emerged as a new class of potent bioactive molecules, implicated in a variety of cellular processes, such as cell differentiation, apoptosis, and proliferation [11]–[13]. Phosphatidylcholine-specific Phospholipase D (PLD) is the enzyme which hydrolyzes phosphatidylcholine, to generate phosphatidic acid (PA) and choline [11], [12]. PA, a potent second messenger by itself, can be dephosphorylated to Diacylglycerol (DAG), or hydrolyzed to Lyso-phosphatidic acid (LPA), by Phosphatidic acid phosphohydrolases and Phospholipase A2 respectively [13]–[17]. Intracellularly, PLD, or its product PA, is known to regulate a variety of homeostatic cellular functions such as membrane trafficking, vesicular transport, cytoskeletal re-organization, cellular migration, proliferation and survival [16]–[19]. A role of PLD in immune cell responses *in vitro* is supported by a variety of studies showing PLD to mediate receptor-activated effector responses, including in phagocytosis (20–21), NADPH-oxidative burst [22], [23], immune cell migration [10], [24]–[25], degranulation [26], [27] and cytokine production [10], [28]. PLD comprises two major isoforms, PLD1 and PLD2, expressed in a wide range of almost all the mammalian tissues [18], [26]. PLD1 has been associated with the activation of monocytes/macrophages, neutrophils and mast cells [10], [23], [29]–[31], whereas PLD2 has been associated with responses in T lymphocytes [32], [33]. However, due to lack of isoform-specific inhibitors for *in vivo* work and knockout mice, the role of PLDs, and indeed of individual PLD isoforms in *in vivo* physiology or pathophysiology remains largely unknown.

Here we show for the first time that TNFα-triggered inflammation *in vivo* can be attenuated by targeting PLD1. We show here that the TNFα-triggered temperature changes, cytokine/chemokine production, vascular permeability, cell adhesion molecule expression and neutrophil and monocyte infiltration into the peritoneal cavity, are inhibited in mice where PLD1 has been knocked down. Thus, our data demonstrate a critical role for PLD1 in TNFα-mediated inflammation.

## Materials and Methods

### Ethics

All the animal experiments performed in this study were conducted by strictly adhering to the guidelines stated by National University of Singapore (NUS) Institutional Animal Care and Use Committee (IACUC). The IACUC ethics committee approved this study under the approved IACUC protocol No: 108/06(A1)08).

### Materials

All materials unless stated otherwise were bought from Sigma-Aldrich.

### Mice

All the *in vivo* experiments were carried out on male BALB/c mice (8–10 weeks old) weighing 20–25 grams. The animals were obtained from the NUS, Sembawang Laboratory Animals Centre and housed in the animal holding unit, NUS, prior to and during the experiments. The animals were housed in appropriate cages with free access to food and water.

### siRNA administration and gene knockdown *in vivo*

The mice were anesthetized and siRNAs were administered via intravenous tail vein injections. Based on our earlier *in vivo* siRNA optimization experiments [34], the dose of synthetic siRNAs injected was 4 µg/mouse of siRNA in a final volume of 100 µl, and repeated three times at 24 hour intervals) to achieve an effective knockdown effect. The siRNA specific for mouse PLD1 used to knockdown mouse PLD1 is (AGAGGUGGUUGAUAGUAAA)dTdT & (UUUACUAUCAACCACCUCU)dTdT. A scrambled siRNA (Allstar negative control) was used as control.

### Gel electrophoresis and Western blot

40 µg of lysate for each sample was resolved on 10% polyacrylamide gels (SDS-PAGE) under denaturing conditions and then electrophoretically transferred to 0.45 µm nitrocellulose membranes. After blocking overnight at 4°C with 5% nonfat milk in Tris-buffered saline and 0.1% Tween 20, and washing, the membranes were incubated with rabbit polyclonal anti-PLD1 or anti-PLD2, and mouse monoclonal anti-α-tubulin (Santa Cruz Biotechnologies, CA) primary antibodies for 2 h at room temperature. The membranes were washed extensively in the washing buffer and incubated with the appropriate horseradish peroxidase-conjugated secondary antibodies (Sigma-Aldrich, Singapore). Bands were visualized using the ECL Western blotting detection system (GE Healthcare, UK).

### TNFα-induced peritonitis model in mice

Acute inflammation in the peritoneal cavity of BALB/c was induced by the intraperitoneal injection (i.p) of recombinant mouse TNFα (PeproTech Inc.Rocky Hill, NJ, USA), at 5 µg/mouse in a final volume of 100 µl. Six mice were used for each group (n = 6 per group) per experiment. All the basal category or control mice were injected (i.p) with 100 µl of sterile saline (PBS). TNFα was administered 24 h after the third dose of the siRNA treatment. The inflammatory parameters measured are explained below.

### Rectal temperature measurements

Temperature changes in the mice as a result of the TNFα-induced inflammatory response were measured rectally using a digital rectal thermometer (Natsume Seisakusyo Co., Tokyo, Japan). The thermometer probe was dipped in oil prior to measurements. The mice were held in a custom built restrainer during the measurements. The probe was inserted into the rectum up to 2 cm deep and held in the same position for 15 seconds until a stable temperature readout was obtained. Temperature was recorded at the times indicated in the figure.

### Peritoneal inflammatory reaction

Acute inflammation in the peritoneal cavity was induced by an i.p. injection of recombinant mouse TNFα (5 µg/mouse in a final volume of 100 µl) into mice either pretreated with siRNA or saline control. At the indicated times, mice were sacrificed, and their peritoneal cavity was washed with 2 ml of ice-cold PBS, 0.1% BSA. The recovered peritoneal lavage fluid was analyzed for vascular permeability, different cell infiltrates and the level of cytokines was measured. Six mice were used for each group per experiment, and the experiments were conducted three times.

### Permeability changes

For permeability analysis, the Evans blue dye at 1% in a final volume of 100 µl of PBS was i.v. administered 10 min before the TNFα or PBS-(vehicle) i.p. administration. At the indicated times, mice were sacrificed, and their peritoneal cavity was washed with 2 ml of ice-cold PBS, 0.1% BSA. The cells were spun down and the OD of the supernatant at 620 nm was measured as an indicator of Evans blue leakage into the peritoneal cavity. Five mice were used for each group per experiment, and the experiments were conducted three times.

### Collection of serum

Following the TNFα challenge for 12 h, whole blood was collected, by cardiac puncture, placed in collection tubes and allowed to clot spontaneously on ice. Once the clot was formed, the serum was gently pipetted out to a fresh tube. This collected serum was further clarified by centrifugation at 3000 rpm for 10 minutes and the supernatant (serum) was collected gently, without disturbing the pellet, and stored at −20°C until use.

### Measurement of cytokine production

The levels of mouse IL-6, MIP-1α and MIP-1β in the peritoneal lavage and serum were measured using mouse IL-6, mouse MIP-1α and mouse MIP-1c OptEIA™ Kit (BD Biosciences, San Jose, CA, USA), following the manufacturer's instructions.

### Histology

For morphological investigation, the peritoneal membranes of mice, 12 h after the TNFα challenge, were carefully dissected out and immersed in 10% formalin fixative for 1 day. The specimens were then dehydrated through an ascending series of ethanol and cleared in toluene, before being embedded in paraffin. The tissue blocks were cut at 4 µm thickness by means of a Leica Rotary Microtome (Model 2165) and processed for Hematoxylin and Eosin (H&E) staining, as described before [34]. Once dried, the sections were observed using a light microscope (Leica DM IRB microscope) and the images were captured using a Leica DC 300F digital camera. The images were analyzed with the Leica IM500 Image Manager software.

### Immunofluorescence of CAMs

Peritoneal membrane sections, prepared as described above, were deparaffinized in two changes of xylene, 5 minutes each. Then they were hydrated in two changes of 100% ethanol for 3 minutes each, 95% and 80% ethanol for 1 minute each and rinsed in distilled water. Then the sections were incubated with 2N HCl solution for 20 minutes, for antigen retrieval. The tissues were marked with a circle using a DAKO pen (DAKO Biotech, Inc. Glostrup, Denmark). Then they were permeablized using 0.2% Saponin for 10 minutes and washed in 1×TBS, 3×5 min. Goat serum (1∶30) was added for 30 min at room temperature and drained with blotting paper. The tissues were incubated with the primary antibody, VCAM1 or ICAM1 (Santa Cruz Biotechnology, Inc., USA), for the respective cell adhesion molecule to be probed overnight in a humidity chamber at 4°C. Then they were washed with TBS, three times for 5 minutes. The corresponding secondary antibody (DAKO Biotech, Inc. Glostrup, Denmark), labeled with FITC, was added at room temperature for 1 hr. The tissues were then washed with TBS, three times for 5 min. Finally the slides were mounted with a cover slip, using fluorsave. The sections were visualized with an inverted fluorescence Leica DM IRB microscope and the images were captured using a Leica DC 300F digital camera. The images were analyzed with the Leica IM500 Image Manager software.

### Statistical analysis

Statistical differences between control and treated groups/samples were calculated using the unpaired Student's *t-*test. Student's *t* test *p* values (*p<0.05 and **p<0.01).

## Results

### In vivo knockdown of PLD1

**Figure 1:**
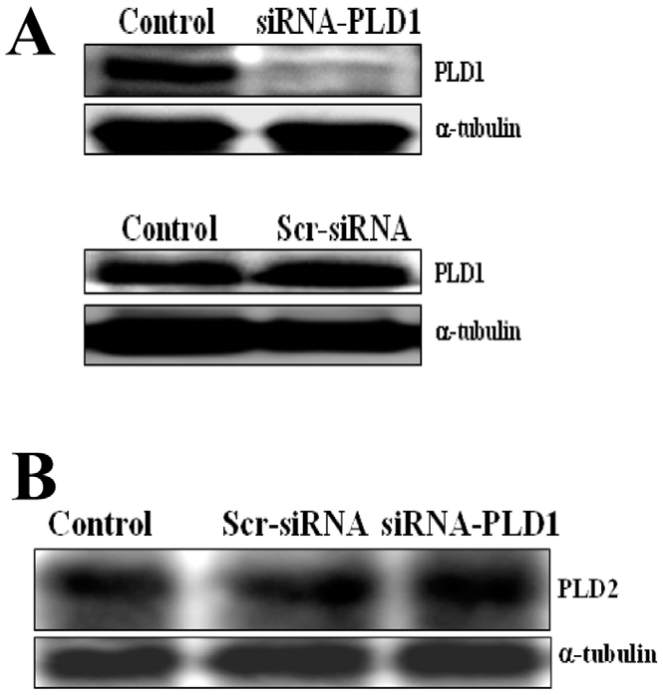
Specific knockdown of PLD1 in BALB/c mice using siRNA. (**A**) Western blot analysis shows the knockdown of murine phospholipase D1 (PLD1) in BALB/c mice PBMNCs, by the use of specific siRNA for PLD1 (4 µg/mouse). (**B**) Western blot analysis shows the expression of murine phospholipase D2 (PLD2) in the same set of samples as indicated. Lysates probed for PLD1 and PLD2 from PBMCs were obtained from: untreated mice which served as control (Control); mice injected with siRNA-PLD1 (siRNA-PLD1); and from mice injected with scrambled siRNA (Scr-siRNA). In addition, α-tubulin was probed for loading control.

We and others have shown that intravenous administration of siRNA is known to result in a significant knockdown of the gene products in mice [32]–[34]. Figure 1a shows a significant decrease in the protein level of murine phospholipase D1 (mPLD1) in peripheral blood leukocytes of mice, following intravenous administration of siRNA (4 µg/mouse) to knockdown mPLD1 (**Fig. 1A**). Since both the PLD isoforms are expressed in BALB/c mice [35], we determined the isoform-specific knockdown of PLD1 by evaluating the expression of PLD2 as well, following the above-mentioned siRNA intravenous administration. The level of mPLD2 expression remained unaffected subsequent to siRNA treatment specific for PLD1 (**Fig. 1B**), indicating the efficiency and specificity of the isoform-specific siRNA knockdown of PLD1 *in vivo*.

### Role of PLD1 in TNFα— induced temperature changes and serum cytokines

**Figure 2:**
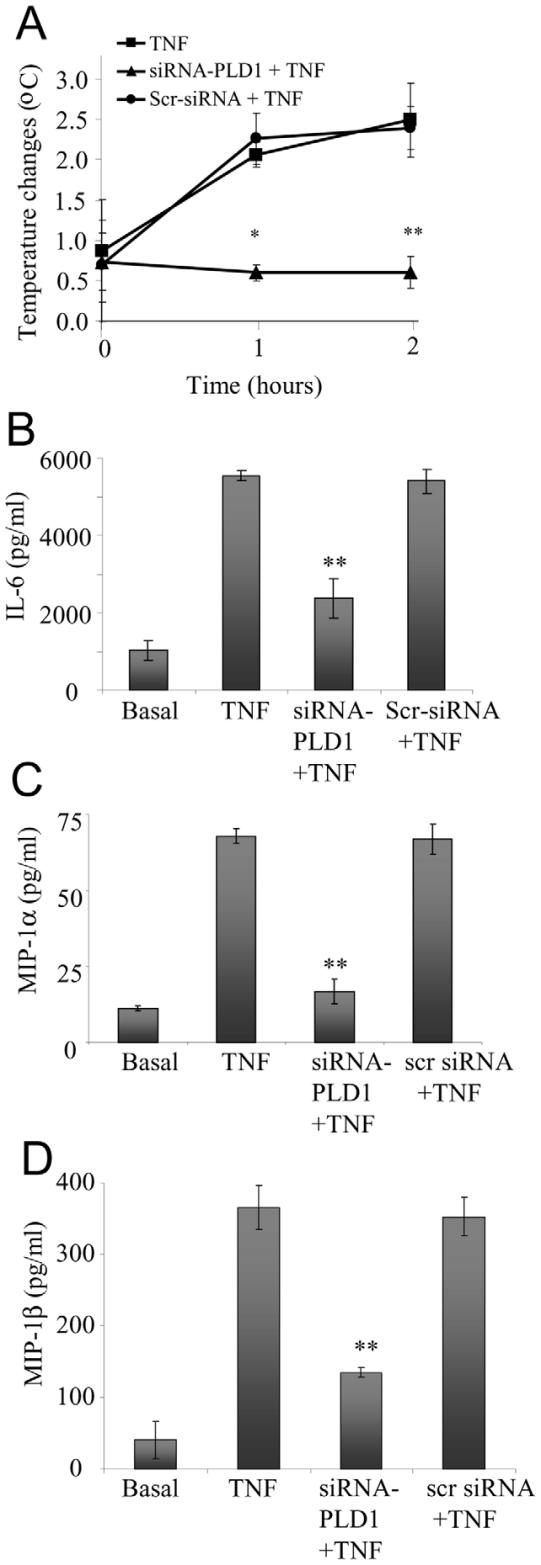
TNFα-induced changes in temperature and serum cytokine levels is dependent on PLD1. (**A**) TNFα-induced temperature changes observed over the time points indicated. TNFα temperature changes in: control mice (TNF); in mice pretreated with the siRNA-PLD1 (siRNA-PLD1+TNF); and in mice pretreated with the scrambled siRNA (Scr-siRNA+TNF). TNFα-induced changes in serum levels of: IL-6 (**B**); MIP-1α (**C**), and MIP-1β (**D**) levels in control mice (Basal); in mice injected with TNFα (TNF); in mice pretreated with the siRNA-PLD1 prior to TNFα administration (siRNA-PLD1+TNF); and in mice pretreated with the scrambled siRNA prior to TNFα administration (Scr-siRNA+TNF). Data showed as means ± SD of triplicate measurements from three different experiments. Student's *t* test *p* values (*p<0.05, **p<0.01). Six mice were used per treatment group per experiment.

One of the characteristic systemic responses related to TNFα administration is pyrexia or increase in body temperature. Therefore, the change in body temperature of mice following TNFα intra-peritoneal administration and the role of PLD1 in this response was evaluated. Mice injected with TNFα developed a marked increase in body temperature in an hour (**Fig. 2A**). This increase in body temperature was considerably reduced in mice pretreated with the PLD1 siRNA. However, the rise in temperature in mice pretreated with the negative control siRNA was identical to that of the TNFα treated mice positive control.

A key event in the inflammatory process is the production of proinflammatory cytokines. TNFα is capable of amplifying its inflammatory response by promoting the generation and release of several proinflammatory cytokines and chemokines. Therefore we investigated the levels of IL-6 (**Fig. 2B**), MIP-1α (**Fig. 2C**) and MIP-1β(**Fig. 2D**) in the serum following TNFα administration. We found there was a significant rise in the levels of these proinflammatory cytokine and chemokines in the serum of mice treated with TNFα (**Fig. 2B–D** **)**. In contrast, an inhibition in this response was observed when the mice were treated with siRNA-PLD1 prior to TNFα injection (Fig. **2B–D**). This indicates the necessity of PLD1 in this response.

### TNFα-triggered vascular permeability and peritoneal cell adhesion molecule expression

**Figure 3:**
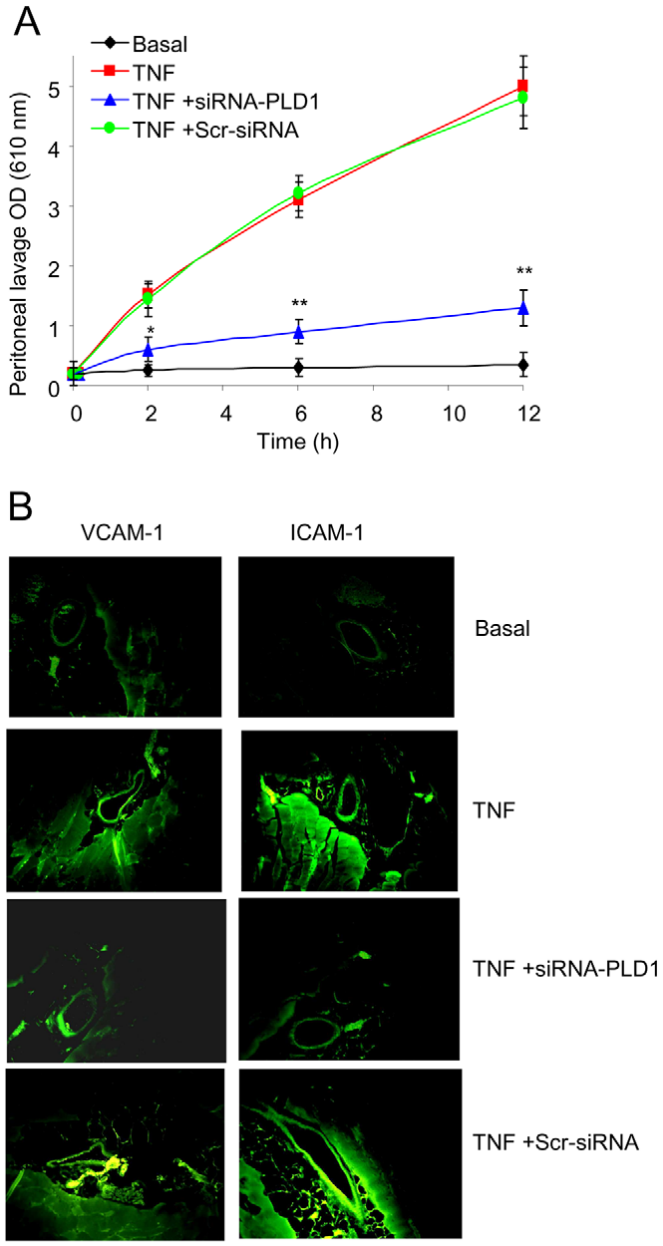
TNFα-induced vascular permeability and VCAM-1 and ICAM-1 expression in peritoneal tissues is dependent on PLD1. (**A**) Peritoneal lavage was collected, and the OD was measured, from mice: untreated following the i.p. injection of PBS (Basal); following the i.p. injection of TNFα (TNF); following the i.p. injection of TNFα in mice pretreated with the siRNA-PLD1 (TNF +siRNA-PLD1); following the i.p. injection of TNFα in mice pretreated with the scrambled siRNA (TNF +Scr-siRNA). Data showed as means ± SD of triplicate measurements from three different experiments. Student's *t* test *p* values (*p<0.05, **p<0.01). Six mice were used per treatment group per experiment. (**B**) VCAM-1 and ICAM-1 expression pattern using immunohistochemistry in peritoneal tissues after 2 h of TNFα administration. The panels indicate the peritoneal tissues from control mice (Basal); from mice injected with TNFα (TNF); from mice pretreated with the siRNA-PLD1 prior to TNFα administration (TNF+siRNA-PLD1); and from mice pretreated with the scrambled siRNA prior to TNFα administration (TNF+Scr-siRNA). Results shown are representative of three different experiments and of multiple sections and fields.

One of the characteristic events in an inflammatory response is the increase in vascular permeability, and vascular cell adhesion molecule expression. Alterations in vascular permeability were determined by i.v. injection of Evans blue dye, which binds to serum proteins and thus can be used to quantify alterations in vascular permeability. Intra-peritoneal injection of TNFα into the peritoneal cavity caused a steady influx of Evans blue into the peritoneal cavity, with a continued increase from 2 to 12 h (**Fig. 3A**).

It is well known that cell adhesion molecules are important mediators in cellular migration and infiltration. Therefore, we evaluated the expression of ICAM1 and VCAM1 on the vascular endothelium, using immunohistochemistry in peritoneal tissues. Intra-peritoneal injection of TNFα caused an increase in the expression of VCAM-1 and ICAM-1 (**Fig. 3B**) on the vascular endothelium. However, in mice pretreated with the siRNA-PLD1, there was a substantial decrease in the TNFα-triggered expression of the cell adhesion molecules (**Fig. 3B**).

### TNFα-triggered peritoneal cytokine and chemokine production

**Figure 4:**
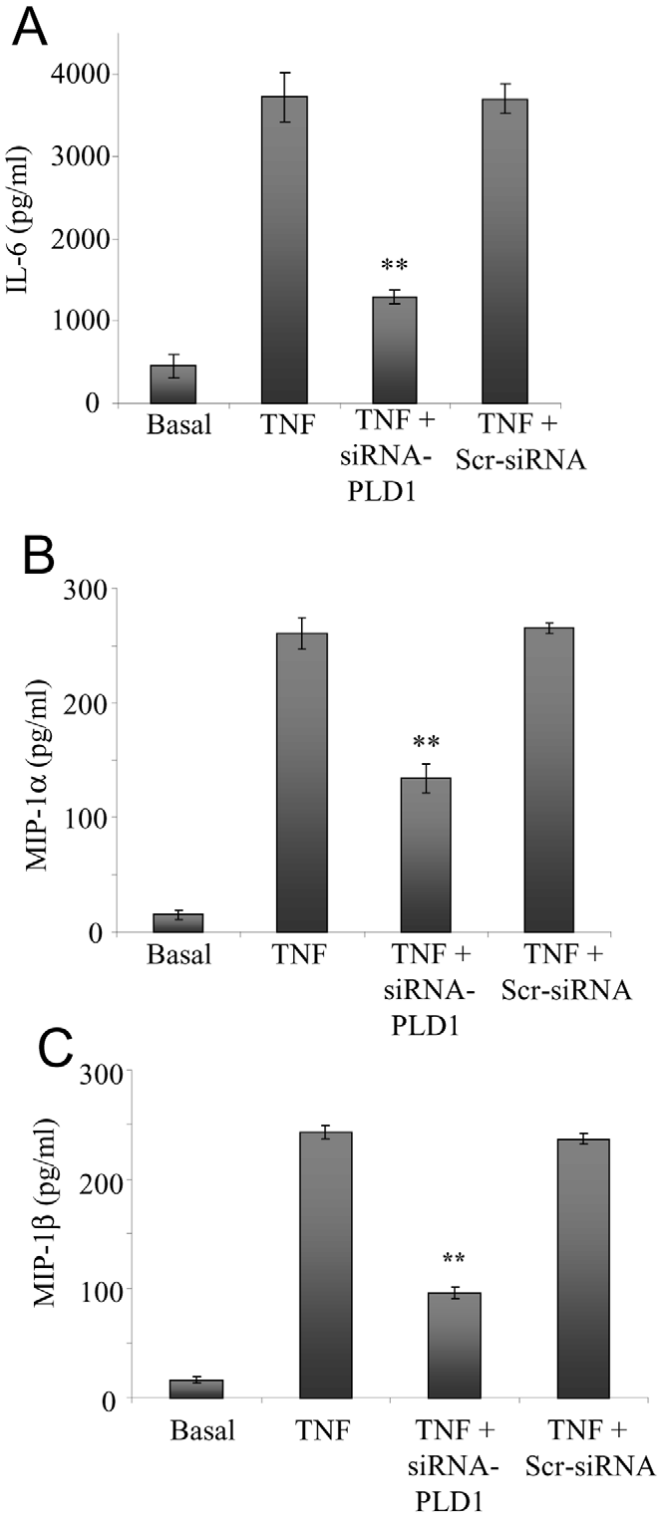
TNFα-induced peritoneal cytokine/chemokine levels. TNFα-induced changes in serum levels of: IL-6 (**A**); MIP-1α (**B**), and MIP-1β (**C**) levels in control mice (Basal); in mice injected with TNFα (TNF); in mice pretreated with the siRNA-PLD1 prior to TNFα administration (siRNA-PLD1+TNF); and in mice pretreated with the scrambled siRNA prior to TNFα administration (Scr-siRNA+TNF). Data showed as means ± SD of triplicate measurements from three different experiments. Student's *t* test *p* values (*p<0.05, **p<0.01). Six mice were used per treatment group per experiment.

A key event in localized inflammatory processes is the production of proinflammatory cytokines and chemokines. Localized cytokines and chemokines amplify the inflammatory response, by promoting vascular permeability and the influx of immune cells into the affected area. Therefore, we investigated the levels of IL-6 (**Fig. 4A**), MIP-1α (**Fig. 4B**) and MIP-1β (**Fig. 4C**) in the peritoneal lavage, following TNFα administration. We found there was a significant rise in the levels of these proinflammatory cytokine and chemokines in the peritoneal lavage of mice treated with TNFα (**Fig. 4A–C**). In contrast, an inhibition in this response was observed when the mice were treated with siRNA-PLD1 prior to TNFα injection (**Fig. 4A–C**). This indicates that PLD1 is required for the localized generation of these proinflammatory mediators.

### TNFα-triggered neutrophil and monocyte infiltration into the peritoneal cavity

**Figure 5:**
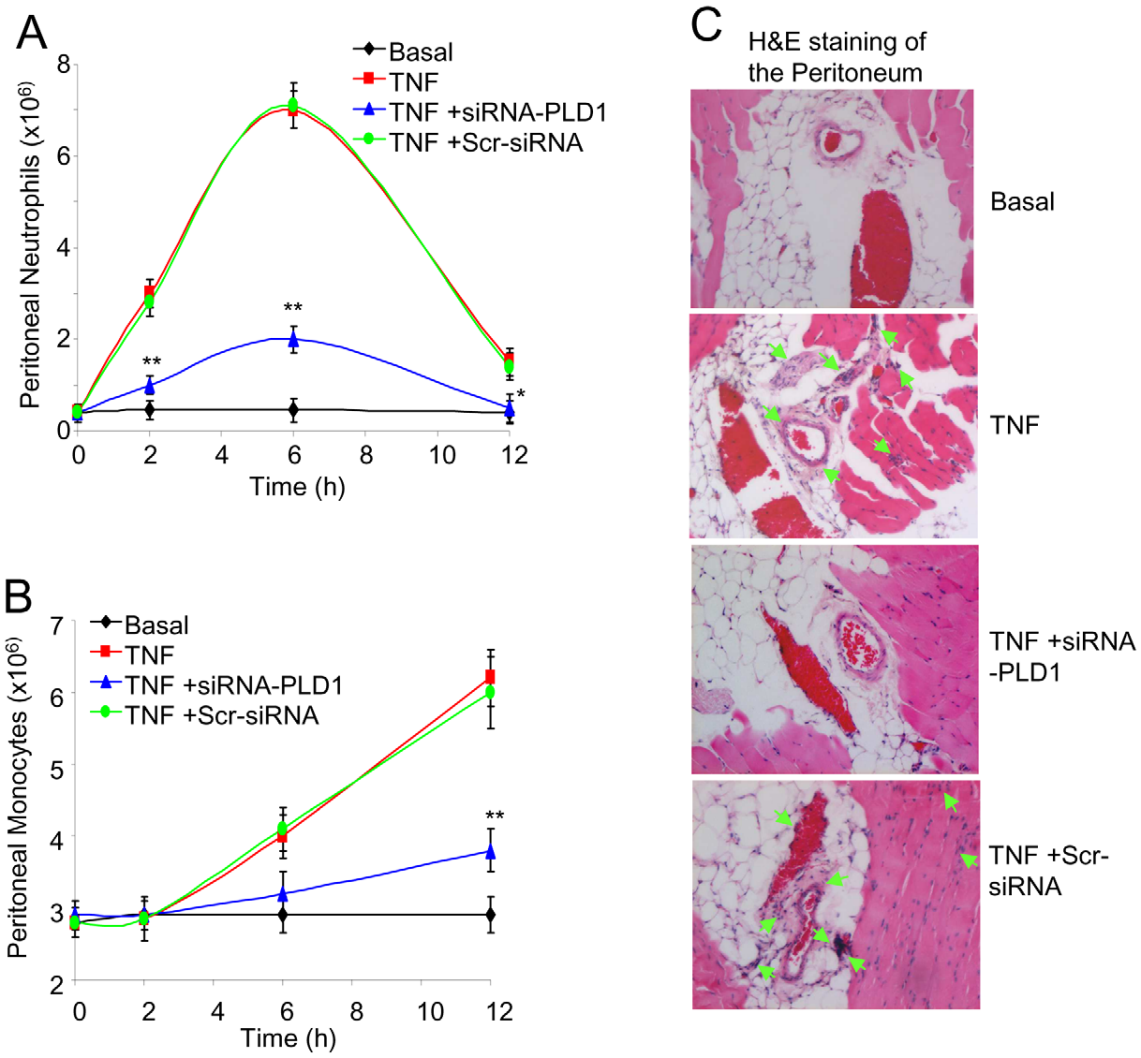
TNFα-induced immune cell infiltration into the peritoneal cavity. (**A**) Neutrophil, and (**B**) monocyte infiltration into peritoneal cavity following TNFα administration for the indicated times, measured in the peritoneal lavage of: control mice injected with saline alone (Basal); of mice injected with TNFα (TNF); of mice pretreated with the siRNA-PLD1 prior to the TNFα administration (TNF+siRNA-PLD1); and of mice pretreated with the scrambled siRNA prior to the TNFα administration (TNF+Scr-siRNA). Data showed as means ± SD of triplicate measurements from three different experiments. Student's *t* test *p* values (*p<0.05, **p<0.01). Six mice were used per treatment group per experiment. (**C**) Haematoxilin and Eosin staining of peritoneal tissues after TNFα administration. The panels indicate the peritoneal tissues from: control saline injected mice (Basal); mice injected with TNFα (TNF); from mice pretreated with the siRNA-PLD1 prior to TNFα administration (TNF+siRNA-PLD1); and from mice pretreated with the scrambled siRNA prior to TNFα administration (TNF+Scr-siRNA). Results shown are representatives of multiple experiments and of multiple sections and fields. Green arrows indicate a high number of cellular infiltrates.

Acute inflammatory responses are characterized by the rapid recruitment of phagocytic cells to the site of inflammation. Intra-peritoneal administration of TNFα induced a significant influx of neutrophils (**Fig. 5A**) and monocytes (**Fig. 5B**) into the peritoneal cavity, compared to that of the untreated controls. This TNFα-triggered increased influx of neutrophils and monocytes into the peritoneal cavity was significantly inhibited in mice pretreated with PLD1 siRNA (**Fig. 5A–B**).

The role of PLD1 in TNFα-triggered migration of leukocytes into the site of inflammation was further evaluated using Haematoxilin and Eosin (H&E) staining of the peritoneal tissues. The peritoneal tissues from mice challenged with TNFα exhibited a marked increase in cellular infiltration (indicated by green arrows) (**Fig. 5C**), when compared to basal levels (**Fig. 5C** top panel). However, this TNFα-triggered increase in cellular infiltration was markedly inhibited in the peritoneal tissues of mice pretreated with the siRNA against PLD1 (**Fig. 5C** third panel). In contrast, the TNFα-induced cellular infiltration was unchanged in mice pretreated with the scrambled siRNA (**Fig. 5C** bottom panel).

Taken together, our data demonstrate a critical role for PLD1 in the inflammatory responses triggered by TNFα *in vivo*.

## Discussion

In this study we have attempted to elucidate some of the molecular mechanisms utilized by TNFα, during the inflammatory response. This is an important area of research, as TNFα is known to be linked to a wide range of inflammatory pathologies.

Inflammation is involved in the maintenance of tissue homeostasis, defense against infection and mediating immune responses. However, a dysregulated or prolonged inflammatory process contributes to tissue injury and morbidity, especially in systemic-acute and chronic inflammatory conditions, such as in sepsis and autoimmune diseases. This leads to the necessity of dampening the inflammatory response. TNFα is well known for its role in host defense against bacterial, viral and parasitic infections. However, aberrant TNFα responses have been associated with a spectrum of inflammatory disorders. Biological agents, antibodies and soluble receptors, which target TNFα actions, are being increasingly used in the management of inflammatory disorders. A range of them are currently licensed as TNFα-blocking agents and are being used in the management of inflammatory diseases, including rheumatoid arthritis, ankylosing spondylitis and Crohn's disease, with a varying degree of success [36]–[38].

However, TNFα blockade has been associated with an increase in susceptibility to bacterial, viral and parasitic infections, including Listeria, Mycobacteria and granulomatous infections [36]–[39]. It has also been found to be associated with the incidence of opportunistic infection, demyelinating syndromes and autoimmune conditions like lupus. A recent report by Jan Lin *et al* [7], has discussed in detail the adverse effects induced by TNFα blockade, which clearly indicates the limitations of the use of such biopharmaceuticals. The lack of responsiveness to certain disorders, susceptibility to infections and resistance in long-term use has increased the search for alternative therapeutic agents.

This study is the first study which validates the *in vivo* relevance of PLD1 in the process of inflammatory responses. Increase in body temperature is a systemic response to infection or inflammation and it is known to be mediated by endogenous pyrogens, including TNFα and IL-6 [40]. Interestingly, it has also been reported that TNFα-induced pyrexia in mice was largely mediated by IL-6 production [41]. Our study shows that TNFα-induced pyrexia was inhibited when PLD1 was knocked down. We have also shown that PLD1 is necessary for TNFα-induced IL-6 production *in vivo*. The production of proinflammatory cytokines are one of the characteristic features of inflammation. Our study shows that PLD1 is essential in a TNFα-induced increase in the levels of proinflammatory cytokines/chemokines, which are important in the amplification of the inflammatory process. Thus, the knockdown of PLD1 *in vivo* prevented the TNFα-triggered increase in IL-6, MIP-1α and MIP-1β, both localized and systemic. IL-6, apart from modulating homeostatic functions like proliferation, differentiation, survival and apoptosis, also plays a major role in the amplification of inflammatory responses [42], [43]. Increased or abnormal IL-6 levels are associated with a plethora of inflammatory conditions, such as inflammatory bowel disease, asthma, multiple myeloma, rheumatoid arthritis and other autoimmune diseases [44]–[49]. MIP-1α and MIP-1β produced in response to inflammatory stimuli also contribute to the inflammatory process by inducing responses such as chemotaxis, degranulation, phagocytosis and eicosanoid generation [50]. Dysregulation of MIP-1 has been associated with inflammatory disorders including arthritis [50], [51]. Thus, blockade of PLD1 may have the potential for reducing the IL-6 and MIP1α/β adverse effects in inflammatory conditions.

The peritoneal Arthus reaction is characterized by acute inflammation that involves the migration of PMN, vascular leakage, and cytokine production in the peritoneal cavity. We report here that the TNFα i.p. administration triggered an inflammatory response that was inhibited in mice where PLD1 had been knocked down. We observed that the TNFα i.p. injection triggered a fast recruitment of neutrophils, later followed by monocytes, into the peritoneal cavity. Vascular permeability was also observed: when we i.v. injected Evans blue prior to TNFα i.p. injection, we could observe a continued influx of the dye into the peritoneum. However, in mice pretreated with the siRNA-PLD1, there was a significant reduction in the TNFα-triggered neutrophil and monocyte infiltration, as well as a marked reduction in the Evans blue influx. We also show here that the i.p. administration of TNFα caused an increase in CAMs and cytokine/chemokine levels in the peritoneal cavity, and that this increase was substantially inhibited in mice pretreated with the siRNA-PLD1.

It is well-established that phagocytic cell infiltration and proinflammatory cytokine production are universal components of a wide range of inflammatory conditions and diseases, such as nephritis [52], arthritis [53], and acute graft rejection [54]. Thus, agents that can inhibit phagocyte infiltration and/or the production of cytokines and chemokines may have wide therapeutic applications in the prevention and treatment of inflammatory diseases. The present study indicates that genetic silencing of *PLD1*, leading to the knockdown of PLD1 expression, very effectively blocked the cytokine/chemokine production, vascular permeability and leukocyte recruitment triggered by TNFα *in vivo*. Interestingly, at the cellular level, it has been reported that upregulation in the expression of ICAM-1, VCAM-1, IL-6 and MIP1α/β is mediated by ERK1/2 and NFκB activities [reviewed in ref. 4]. This is in agreement with our earlier finding that TNFα-triggered ERK1/2 and NFκB activities and proinflammatory cytokine/chemokine production are downstream of PLD1 activation [10].

Taken together, these observations suggest a potential role for PLD1 in the TNFα-triggered proinflammatory responses *in vivo*. However, it is possible that the knockdown of PLD1 has an effect not only on the TNFα-mediated signaling, but also on other receptors that may be stimulated as secondary events, following TNFα-triggered responses.

The results presented here are relative to changes in mice during *i.p.* injection of TNFα, compared to mice that have been injected with saline alone. Whether the observed changes in the inflammatory responses triggered by TNFα, under these experimental conditions, are representative of a pathological state, is not currently known. However, these observations regarding the molecular basis of the inflammatory response are likely to improve our knowledge of the mechanisms, by which TNFα may contribute to the overall activation of the immune response. Thus, blockade of PLD1 has potential clinical implications for improving not only acute inflammatory conditions, but also other inflammatory diseases where TNFα plays a role.
